# The anterior insula and its projection to amygdala nuclei modulate the abstinence-exacerbated expression of conditioned place preference

**DOI:** 10.1007/s00213-023-06499-0

**Published:** 2023-11-27

**Authors:** Andrés Agoitia, Apolinar Cruz-Sanchez, Israela Balderas, Federico Bermúdez-Rattoni

**Affiliations:** División de Neurociencias, Instituto de Fisiología Celular, Universidad Nacional Autónoma de México, 04510 Mexico City, Mexico

**Keywords:** Anterior insular cortex, Amygdala, Conditioned place preference, Incubation of drug craving, Abstinence, Amphetamine, Incentive salience, Rewarding contextual memory

## Abstract

**Rationale:**

Relapse into substance use is often triggered by exposure to drug-related environmental cues. The magnitude of drug seeking depends on the duration of abstinence, a phenomenon known as the incubation of drug craving. Clinical and preclinical research shows that the insular cortex is involved in substance use disorders and cue-induced drug seeking. However, the role of the insula on memory retrieval and motivational integration for cue-elicited drug seeking remains to be determined.

**Objectives:**

We investigated the role of the anterior insular cortex (aIC) and its glutamatergic projection to amygdala nuclei (aIC-AMY) on the expression of conditioned place preference (CPP) during early and late abstinence.

**Methods:**

Male adult C57BL/6J mice underwent amphetamine-induced CPP, and their preference was tested following 1 or 14 days of abstinence. aIC and aIC-AMY functional role in CPP expression was assessed at both abstinence periods by employing optogenetic silencing and behavioral pharmacology.

**Results:**

Compared to a single day, an exacerbated preference for the amphetamine-paired context was observed after 14 days of abstinence. Photoinhibition of either aIC or aIC-AMY projection reduced CPP expression following late but not early abstinence. Similarly, the antagonism of aIC NMDA receptors reduced CPP expression after 14 days of abstinence but not 1 day.

**Conclusions:**

These results suggest that aIC and its glutamatergic output to amygdala nuclei constitute critical neurobiological substrates mediating enhanced motivational cue reactivity during the incubation of amphetamine craving rather than contextual memory recall. Moreover, cortical NMDA receptor signaling may become sensitized during abstinence, ultimately modulating disproportioned drug seeking.

**Supplementary Information:**

The online version contains supplementary material available at 10.1007/s00213-023-06499-0.

## Introduction

Under certain conditions, chronic substance use may lead individuals to develop substance use disorders (SUDs), a recurrent pattern of substance use that persists despite experiencing adverse consequences (American Psychiatric Association 2013). Colloquially referred to as drug addictions, SUDs constitute a worldwide public health issue. In 2021, the global prevalence of SUDs (excluding alcohol and tobacco) was estimated at 39.5 million, approximately 13.3% of people who use drugs (United Nations Office on Drugs and Crime 2023). Moreover, in 2019, SUDs accounted for 18.1 million disability-adjusted life years, as much as 59% of the total amount attributed to substance use.

Relapse to substance use constitutes a core criterion for SUDs and is commonly precipitated by exposure to drug-related environmental cues due to associative learning (Crombag et al. 2008). It has been established that the magnitude of cue-induced drug seeking depends on the length of abstinence over a period that extends from the first weeks to several months after the onset of drug withdrawal (Grimm et al. 2001). This associative phenomenon, termed *incubation of drug craving*, has been described in animal models following the chronic intake of a variety of substances such as stimulants, opioids, ethanol, and nicotine (Altshuler et al. 2020; Pickens et al. 2011; Wolf 2016). A similar pattern of cue-elicited craving enhanced by abstinence has been reported in tobacco smokers and patients with methamphetamine or alcohol dependency, often after acute withdrawal symptoms had faded off (Bedi et al. 2011; Li et al. 2015a; Wang et al. 2013). Such an exacerbated display of conditioned drug seeking across time reflects an intensified motivational state (craving) elicited by drug-associated cues, the neurobiological basis of which is not fully understood. However, incubation of drug craving has been proposed to result from the progressive alteration of mechanisms that control neuronal activity within several regions of the brain reward system, including ventral tegmental area (VTA), nucleus accumbens (NAc), amygdala, and cortical structures (Altshuler et al. 2020; Pickens et al. 2011; Wolf 2016).

The insular cortex, a temporal lobe region lying deep within the lateral sulcus, has been related to gustatory processing, recognition memory, bodily interoception (Bermúdez-Rattoni 2004, 2014; Gogolla 2017), and more recently SUDs and drug seeking (Droutman et al. 2015; Ibrahim et al. 2019; McGregor and LaLumiere 2023; Naqvi et al. 2014). In this regard, prospective and retrospective clinical studies (Abdolahi et al. 2015; Naqvi et al. 2007; Suñer-Soler et al. 2012) observed long-term heavy tobacco smokers to undergo sudden smoking cessation following stroke-induced damage to the insula. Interestingly, these patients experienced reduced urges that frequently motif relapse and remained abstinent more easily.

Preclinical studies further indicate that the insular cortex plays a critical role in drug self-administration (Forget et al. 2010; Hollander et al. 2008; Jaramillo et al. 2018; Pushparaj et al. 2015), cue-induced reinstatement of drug seeking (Arguello et al. 2017; Campbell et al. 2019; Cosme et al. 2015; Di Pietro et al. 2006; Forget et al. 2010; Ghareh et al. 2022; Pushparaj et al. 2015), acquisition and expression of drug-induced conditioned place preference (CPP) (Contreras et al. 2007; Li et al. 2013; Loney et al. 2021; Scott and Hiroi 2011; Wu et al. 2014; Zhang et al. 2019), and maintenance of rewarding contextual memory (Contreras et al. 2012; Gil-Lievana et al. 2020; Scott and Hiroi 2011). However, the mentioned approaches were often performed following the extinction of seeking responses over extensive periods, thus including the motivational impact of abstinence to memory-driven drug seeking. Consequently, the specific process by which the insular cortex influences cue-elicited drug seeking remains to be determined.

Moreover, reciprocal anatomical connections exist between the insula and the amygdala (Gehrlach et al. 2020), a brain region causally linked to the incubation of drug craving (Altshuler et al. 2020; Pickens et al. 2011). Recent studies have described an association between the incubation of drug craving and the activity of the anterior insular cortex (aIC) and the amygdala (Reiner et al. 2020; Venniro et al. 2017, 2018). A pharmacological and chemogenetic approach showed that the activity of aIC and its glutamatergic efference to the central nucleus of the amygdala (CeA) partially mediates cue-induced drug seeking following extended abstinence (Venniro et al. 2017). However, the functional role of aIC and its output to the amygdala have not been thoroughly investigated during both early and late abstinence periods.

In the present study, to elucidate the role of the aIC and its projection to the amygdala (aIC-AMY) on the motivational and memory processing involved in cue-induced drug seeking, we performed a series of experiments using CPP, a Pavlovian-based paradigm used to assess the motivational value of drug-associated contextual cues, as has been previously demonstrated to reflect the incubation of drug craving (Li et al. 2008). We first sought to generate an amphetamine-induced CPP procedure that captured the incubation of amphetamine cravings between 1 and 14 days of abstinence. Next, we examined the effect of optogenetic photoinhibition of the aIC and aIC-AMY projection on CPP expression during both periods of abstinence. Furthermore, although NMDA receptors (NMDAR) signaling within aIC has been shown to exert a relevant role in the maintenance of reward-related contextual memory (Contreras et al. 2012; Gil-Lievana et al. 2020), its role on CPP expression has not been sufficiently studied. Based on previous reports that linked the incubation of drug craving with a progressive sensitization of glutamatergic transmission (Caprioli et al. 2015; Lu et al. 2007), specifically NMDAR-mediated signaling within target regions of the brain reward system (Li et al. 2008; Lu et al. 2005), we evaluated the pharmacological blockade of aIC NMDAR on CPP expression at both periods of abstinence.

## Methods and materials

### Subjects

Male adult C57BL/6J mice (8–12 weeks, ≈ 20–28 g body weight) were obtained from the Institute for Cellular Physiology Animal Core. Mice were housed individually and maintained on a 12-h light/dark cycle with a temperature of 22 ± 2 °C and had access to food and water ad libitum. All procedures were approved by the Institutional Animal Care and Use Committee (FBR125-18) and followed the Official Mexican Standard NOM-062-ZOO-1999.

### Drugs

D-amphetamine hemisulfate salt (Sigma-Aldrich, USA) was dissolved to a 0.3 mg/mL concentration in sterile saline. Competitive NMDAR antagonist DL-2-amino-5-phosphonopentanoic acid (AP5, Tocris Bioscience, GBR) was dissolved at 10 μg/μL in sterile saline.

### Viral vectors

Adeno-associated viral vectors AAV5-CamKIIα-eNpHR3.0-eYFP (5.2 × 10^12^ vg/mL) and AAV5-CamKIIα-eYFP (5.1 × 10^12^ vg/mL) were obtained from the University of North Carolina, Gene Therapy Center Vector Core (USA). Upon arrival, vectors were stored in aliquots at − 80 °C until use.

### Stereotaxic surgery

Subjects were anesthetized with isoflurane gas (induction 4%, maintenance 0.5–1.5%, ViP 3000 Matrix, Midmark, USA) and placed on a stereotaxic apparatus (Stoelting Co, USA) with a head holder (Kopf Instruments, USA). A small incision in the scalp was made, and the skull surface was adjusted to the horizontal plane. Small holes were drilled, and a volume of 0.4 μL/side of eNpHR3.0 or eYFP vectors was microinjected bilaterally through a calibrated borosilicate micropipette (5 μL, Drummond, USA) into the aIC (+ 1.9 mm AP, ± 2.8 mm ML, − 2.4 mm DV from dura) and kept in place for 10 min to allow complete diffusion. Subsequently, optical fibers (200 μm core, 0.22NA) embedded within 1.25-mm-wide zirconia ferrules were bilaterally implanted 0.4 mm above aIC or amygdala (− 1.2 mm AP, ± 2.7 mm ML, − 4.3 mm DV from bregma). For pharmacological experiments, 7 mm 23 G stainless steel guide cannulae (Small Parts Inc, USA) were bilaterally implanted above aIC (+ 1.6 mm AP, ± 3.0 mm ML, − 3.0 mm DV from bregma), and removable stylets were placed to maintain patency. Ferrules and cannulae were anchored to the skull surface with dental adhesive and dental acrylic cement. All coordinates were obtained from the Allen reference atlas of the mouse brain (Allen Institute for Brain Science).

### Conditioned place preference

We custom-designed four CPP apparatuses consisting of rectangular acrylic arenas (40 × 20 × 35 cm high) divided by half through a guillotine door into equal-sized square chambers. Each chamber displayed a combination of visual and tactile contextual cues, counterbalanced across arenas. Visual wall cues consisted of a monochromatic pattern of either black and white horizontal stripes or black circles over a white background. Tactile floor cues consisted of diagonally carved straight grooves or a hexagonal grid. Subjects were habituated to handling by the experimenter for 3–5 consecutive days before conditioning. Tests were conducted during the light phase in a sound-attenuated room, with constant white noise and dim lighting above CPP apparatuses. Between trials, CPP apparatuses were thoroughly cleaned with a 70% ethanol solution.

**Fig. 1:**
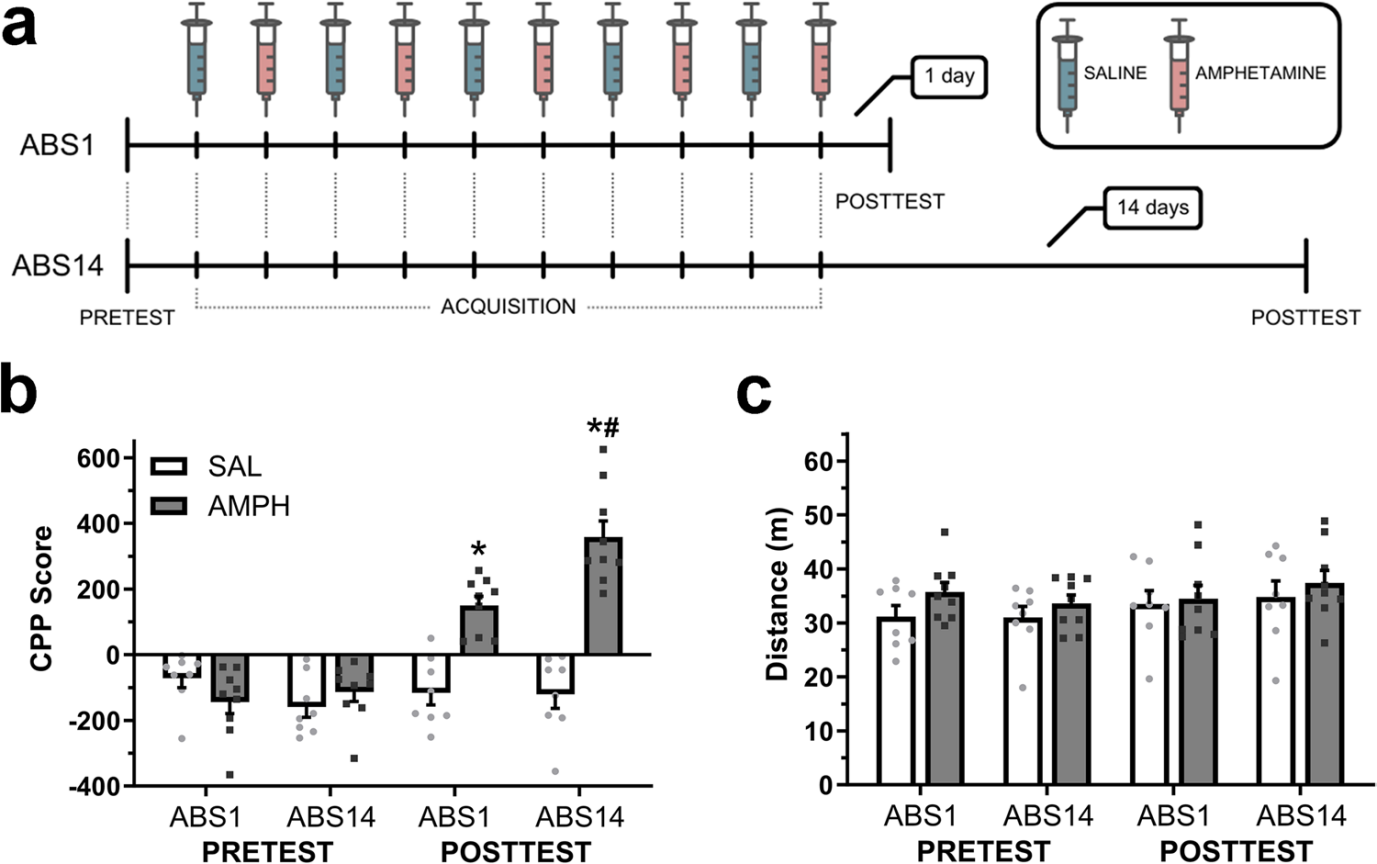
Abstinence-exacerbated CPP expression at 14 days following chronic amphetamine treatment. **a** Schematic of CPP procedure. **b** Baseline preference and CPP expression following early or late abstinence. *p < 0.001 vs. baseline preference, #*p* < 0.01 vs. ABS1. **c** Motor activity during place preference tests

The general CPP procedure (Fig. 1a) was carried out through the following three phases: (1) PRETEST—subjects were allowed to freely explore both chambers for 15 min to determine baseline place preference; to avoid a potential ceiling-effect, we used a biased procedure in which amphetamine was paired to the initially non-preferred chamber, while saline was paired to the preferred chamber. (2) CPP acquisition—on the ten subsequent conditioning days, each subject received daily alternated intraperitoneal injections of either saline (10 mL/kg) or amphetamine (3.0 mg/kg). Immediately after each injection, mice were confined into the corresponding chamber for 30 min. (3) POSTTEST—CPP expression was assessed on a between-subjects design, 1 or 14 days after the last pairing session, on a drug-free state identical to the baseline preference test for 15 min. Time spent on each chamber and distance traveled were obtained using ANY-maze software (Stoelting Co, USA). The CPP score was defined as the difference in time(s) spent in the amphetamine-paired chamber compared to time spent in the saline-paired chamber.

### Optogenetic photoinhibition

Tests were performed 3–5 weeks after surgery to allow opsin expression. Light transmission was achieved using optical tethers, consisting of a diode-pumped solid-state 532 nm laser (150 mW; OEM Laser Systems, USA) coupled to a 62.5 μm core, 0.22NA optic fiber patch cord (Thorlabs, USA), and connected to a single-channel optical rotary joint (Doric Lenses, CAN) before a 50:50 split optical coupler. Light intensity output was adjusted to ≈ 10 mW using a power meter (Thorlabs, USA). Both output fibers were then connected to the implanted ferrules on the subject through a ceramic sleeve to deliver a continuous light pulse for the 15-min duration of the CPP expression test.

### Behavioral pharmacology

Twenty minutes before CPP expression tests, stylets were carefully removed, and a 0.5 μL/side volume of saline or AP5 was microinfused bilaterally into aIC, using 30 G injectors that protruded 1 mm below cannulae tips. Injectors were connected through polyethylene tubing to 10-μL Hamilton syringes (Hamilton Co, USA) driven by a micro-infusion pump (Cole-Parmer Instruments, USA) at a 0.3 μL/min rate. Injectors were kept in place for an additional minute to allow complete diffusion, after which stylets were put back in place.

### Experiment 1 examines the impact of abstinence on the amphetamine-induced conditioned place preference

To assess whether the length of abstinence affects the magnitude of amphetamine-induced CPP, subjects followed a daily schedule of alternated acquisition sessions for 10 days, wherein systemic injections of saline (10 mL/kg, i.p.) or amphetamine (3.0 mg/kg, i.p.) were paired to corresponding chambers for 30 min, based on their initial preference. Subsequently, CPP expression of amphetamine-treated subjects was assessed during a 15-min cue-exposure test performed 1 or 14 days after the last acquisition session (AMPH; ABS1 *n* = 9, ABS14 *n* = 9). Control groups exclusively treated with saline during the entire pairing schedule were tested following the same periods (SAL; ABS1 *n* = 8, ABS14 = 8). In addition, to evaluate whether the abstinence-exacerbated CPP expression could be countered by recent drug exposure, separate groups of amphetamine-treated mice received a non-contingent systemic injection of saline (10 mL/kg, *n* = 9) or amphetamine (3.0 mg/kg, *n* = 9) on its home-cage, 24 h before the CPP expression test performed 14 days after the last acquisition session.

### Experiment 2 investigated the effect of aIC photoinhibition on abstinence-exacerbated CPP expression

To determine whether aIC activity contributes to CPP expression following different abstinence periods, mice were bilaterally injected with a virus encoding a light-driven chloride pump halorhodopsin (eNpHR3.0) and enhanced yellow fluorescent protein (eYFP) or eYFP alone into the aIC, and optic fibers were implanted above the region. Subjects followed the previously described amphetamine-induced CPP procedure wherein CPP expression was evaluated 1 or 14 days after the last acquisition session (eYFP; ABS1 *n* = 9, ABS14 *n* = 10 | eNpHR3.0; ABS1 *n* = 10, ABS14 *n* = 9). During the 15-min cue-exposure test, a 532-nm continuous pulse adjusted to ≈ 10 mW was delivered into aIC through optical tethers.

### Experiment 3 investigated the effect of aIC-AMY photoinhibition on abstinence-exacerbated CPP expression

To further examine whether aIC-AMY glutamatergic projection modulates CPP expression following different abstinence lengths, subjects were bilaterally injected with eYFP or eNpHR3.0 viral vectors into the aIC, and optic fibers were implanted above AMY. Subjects underwent the amphetamine-induced CPP procedure, and CPP expression was evaluated after 1 or 14 days of abstinence (eYFP; ABS1 *n* = 10, ABS14 *n* = 9 | eNpHR3.0; ABS1 *n* = 10, ABS14 *n* = 9). The above-mentioned parameters were similarly used for aIC-AMY photoinhibition during the cue-exposure test.

### Experiment 4 evaluated the pharmacological antagonism of aIC NMDAR on abstinence-exacerbated CPP expression

To determine whether aIC NMDAR signaling is involved in CPP expression following different abstinence periods, subjects were bilaterally implanted with guide cannulae above aIC. The amphetamine-induced CPP procedure was conducted, and CPP expression was tested following 1 or 14 days of abstinence. Twenty minutes before cue-exposure, saline (ABS1 *n* = 11, ABS14 *n* = 11) or AP5 (ABS1 *n* = 12, ABS14 *n* = 10) were microinfused into aIC.

### Histology and immunofluorescence

Subjects received a dose of sodium pentobarbital (75 mg/kg, i.p.) and were transcardially perfused with 0.9% saline, followed by 4% paraformaldehyde (PFA) diluted on a sodium phosphate-buffered solution (0.1 M, pH 7.4). Brains were extracted and post-fixed on 4% PFA overnight at 4 °C and later transferred to a 30% sucrose solution until they sank. Fourty-micrometer coronal sections were obtained using a cryostat (CM1520, Leica Biosystems, DEU) and stored at 4 °C in trizma-base saline buffer (TBS; 150 mM NaCl, Sigma-Aldrich; 0.1 M trizma-base, Sigma-Adrich; pH 7.4). To verify correct cannulae placement, sections were mounted on gelatin-coated slides, stained with cresyl-violet, and observed using an optical microscope. To verify correct fiber placement (Fig. S1a-b) and opsin expression on aIC, free-floating sections were treated with 4′,6-diamidino-2-phenylindole (DAPI), mounted on silanized slides with Dako fluorescence medium (Agilent, USA), and confocal fluorescence images were obtained with a laser-scanning confocal microscope (LSM 800, Zeiss, DEU). Immunofluorescence was performed on aIC-AMY projection: sections were incubated overnight at 4 °C with chicken anti-GFP primary antibody (1:1000, Millipore, USA) on TBS with 0.1% Triton (TBST; 150 mM NaCl, Sigma-Aldrich; 0.1 M trizma-base, Sigma-Adrich; 0.1% Triton X-100, Sigma-Aldrich; pH 7.4) and 5% bovine serum albumin (BSA, Sigma-Aldrich), followed by 2 h incubation at room temperature with goat anti-chicken IgG secondary antibody conjugated with Alexa488 (1:500, Invitrogen, USA) on TBST with 5% BSA, and later counterstained with DAPI. A quantitative analysis was performed on CeA and the basolateral nucleus of the amygdala (BLA) to measure eYFP positive terminals from aIC. Nine confocal images from a representative subject were analyzed (− 0.9 to − 1.6 AP). Images were captured with a × 20 magnification on the *Z*-axis. Using an automated protocol in ImageJ, a square proportion (320 × 320 pixels) was extracted for CeA and BLA, and a binarization threshold was applied ([80, 255]; “BlackBackground”) to measure the mean intensity of eYFP by region of interest.

### Statistics

Data analysis was performed using SPSS Statistics 17.0 (IBM, USA). For CPP expression, three-way mixed ANOVA was performed to compare the main effects of within-subjects “Conditioning,” between-subjects “Group” and “Abstinence,” and their interaction on CPP scores and motor activity during tests. Two-way mixed ANOVA followed significant interaction effects. To simplify the analysis of significant interactions for optogenetics and behavioral pharmacology experiments, posttest CPP scores were considered on a two-way factorial ANOVA to compare the main effects of “Group,” “Abstinence,” and their interaction effects. Bonferroni multiple comparisons were used to identify specific differences among groups. For CPP acquisition, three-way mixed ANOVA was conducted to compare the main effects of within-subjects “Pairing session,” between-subjects “Group,” and “Abstinence,” as well as their interaction on motor activity during acquisition sessions corresponding to the first and last exposure to amphetamine. Significant interaction effects led to subsequent two-way mixed ANOVA. For aIC-AMY projection quantification, eYFP mean intensity between CeA and BLA was compared through a two-tailed paired Student *t*-test. Data represented in all graphs were integrated using Prism 8.0 (GraphPad Software, USA) and are expressed as mean + SEM.

## Results

### Effect of abstinence on amphetamine-induced conditioned place preference

To determine the effect of abstinence length on the magnitude of amphetamine-induced CPP, we compared the CPP scores between baseline preference and cue-exposure tests performed 1 or 14 days after the last acquisition session (Fig. 1b). A three-way mixed ANOVA yielded statistically significant main effects of Conditioning (*F*_(1, 30)_ = 57.18, *p* < 0.001) and Group (*F*_(1, 30)_ = 48.29, *p* < 0.001), but not for Abstinence (*F*_(1, 30)_ = 2.05, *p* = 0.16). Moreover, multiple significant interaction effects were detected between Conditioning by Group (*F*_(1, 30)_ = 59.10, *p* < 0.001), Conditioning by Abstinence (*F*_(1, 30)_ = 6.65, *p* < 0.05), and Group by Abstinence (*F*_(1, 30)_ = 10.23, *p* < 0.01). Two-way mixed ANOVA for each treatment group resulted in significant main effects of Conditioning for amphetamine-treated subjects (*F*_(1, 16)_ = 93.23, *p* < 0.001), Abstinence (*F*_(1, 16)_ = 13.22, *p* < 0.01), and a significant interaction between these factors (*F*_(1, 16)_ = 4.96, *p* < 0.05). Bonferroni multiple comparison analysis showed a significant increase in CPP scores after amphetamine conditioning for both ABS1 (*p* < 0.001) and ABS14 groups (*p* < 0.001) when compared to their initial preference. Additionally, posttest CPP scores for ABS14 were significantly higher (*p* < 0.01) than ABS1. No differences between initial sessions were detected. For saline-treated subjects, no significant main effects were found for Conditioning (*F*_(1, 14)_ = 0.01, *p* = 0.91), Abstinence (*F*_(1, 14)_ = 1.27, *p* = 0.27), or interaction between these factors (*F*_(1, 14)_ = 2.02, *p* = 0.17). Since CPP relies on intact motor activity, we compared the distance traveled during tests (Fig. 1c). A three-way mixed ANOVA found no significant main effects for Conditioning (*F*_(1, 30)_ = 3.54, *p* = 0.07), Group (*F*_(1, 30)_ = 1.89, *p* = 0.17), Abstinence (*F*_(1, 30)_ = 0.06, *p* = 0.80), or any interaction between these factors.

These results demonstrate that contrary to saline, amphetamine treatment produced a robust increase in the time spent in the paired chamber, effectively reversing initial preference. Moreover, following amphetamine treatment, CPP expression was notably higher after 14 days of abstinence than after 1 day (Fig. 1b), with no substantial differences in motor activity between tests or abstinence periods (Fig. 1c).

To evaluate whether CPP expression may have been affected by differences during conditioning between abstinence groups, we compared the distance traveled during acquisition sessions corresponding to the first (AMPH1) and last (AMPH5) exposure to amphetamine (Fig. S2a). A three-way mixed ANOVA yielded statistically significant main effects of the Pairing session (*F*_(1, 30)_ = 44.09, *p* < 0.001), Group (*F*_(1, 30)_ = 88.46, *p* < 0.001), and a significant interaction between these factors (*F*_(1, 30)_ = 33.49, *p* < 0.001). No significant main effects were found for Abstinence (*F*_(1, 30)_ = 0.23, *p* = 0.63) or any other interaction. Two-way mixed ANOVA for each treatment group resulted in a significant main effect of the Pairing session for amphetamine-treated mice (*F*_(1, 16)_ = 49.28, *p* < 0.001) with no significant main effect of Abstinence (*F*_(1, 16)_ = 0.02, *p* = 0.88) or interaction between these factors (*F*_(1, 16)_ = 0.93, *p* = 0.34). For saline-treated mice, no significant main effects were found for the Pairing session (*F*_(1, 14)_ = 1.42, *p* = 0.25), Abstinence (*F*_(1, 14)_ = 0.49, *p* = 0.49), or interaction between these factors (*F*_(1, 14)_ = 0.00, *p* = 0.98).

These results demonstrate that amphetamine treatment significantly increased motor activity during acquisition, compared to saline. Additionally, motor sensitization developed due to chronic amphetamine exposure, with no differences between abstinence periods (Fig. S2a).

To further evaluate whether the abstinence-exacerbated CPP expression could be countered by recent non-contingent drug exposure, we administered conditioned subjects either saline or amphetamine in their home-cage, 24 h before the cue-exposure test performed 14 days after the last acquisition session (Fig. S3a). Two-way mixed ANOVA yielded a significant main effect of Conditioning (*F*_(1, 16)_ = 198.67, *p* < 0.001), with no significant main effect of Group (*F*_(1, 16)_ = 0.60, *p* = 0.44) or interaction between these factors (*F*_(1, 16)_ = 2.64, *p* = 0.12). Regarding motor activity during preference tests (Fig. S3b), two-way mixed ANOVA found no significant main effects of Conditioning (*F*_(1, 16)_ = 0.74, *p* = 0.40), Group (*F*_(1, 16)_ = 0.23, *p* = 0.63), or interaction between these factors (*F*_(1, 16)_ = 0.02, *p* = 0.86). Two-way mixed ANOVA of motor activity during acquisition (Fig. S2b) showed a significant main effect of the Pairing session (*F*_(1, 16)_ = 52.56, *p* < 0.001), with no significant main effect of Group (*F*_(1, 16)_ = 0.06, *p* = 0.79) or interaction between these factors (*F*_(1, 16)_ = 0.15, *p* = 0.69).

These results indicate that both groups displayed higher CPP scores after amphetamine conditioning than their corresponding baseline preference. Moreover, no differences in CPP expression were observed following a non-contingent amphetamine dose administered 24 h before the late abstinence test (Fig. S3a). Motor sensitization developed following chronic amphetamine exposure similarly between groups (Fig. S2b), and no differences in motor activity were observed across tests or between groups (Fig. S3b).

### aIC photoinhibition on abstinence-exacerbated CPP expression

**Fig. 2:**
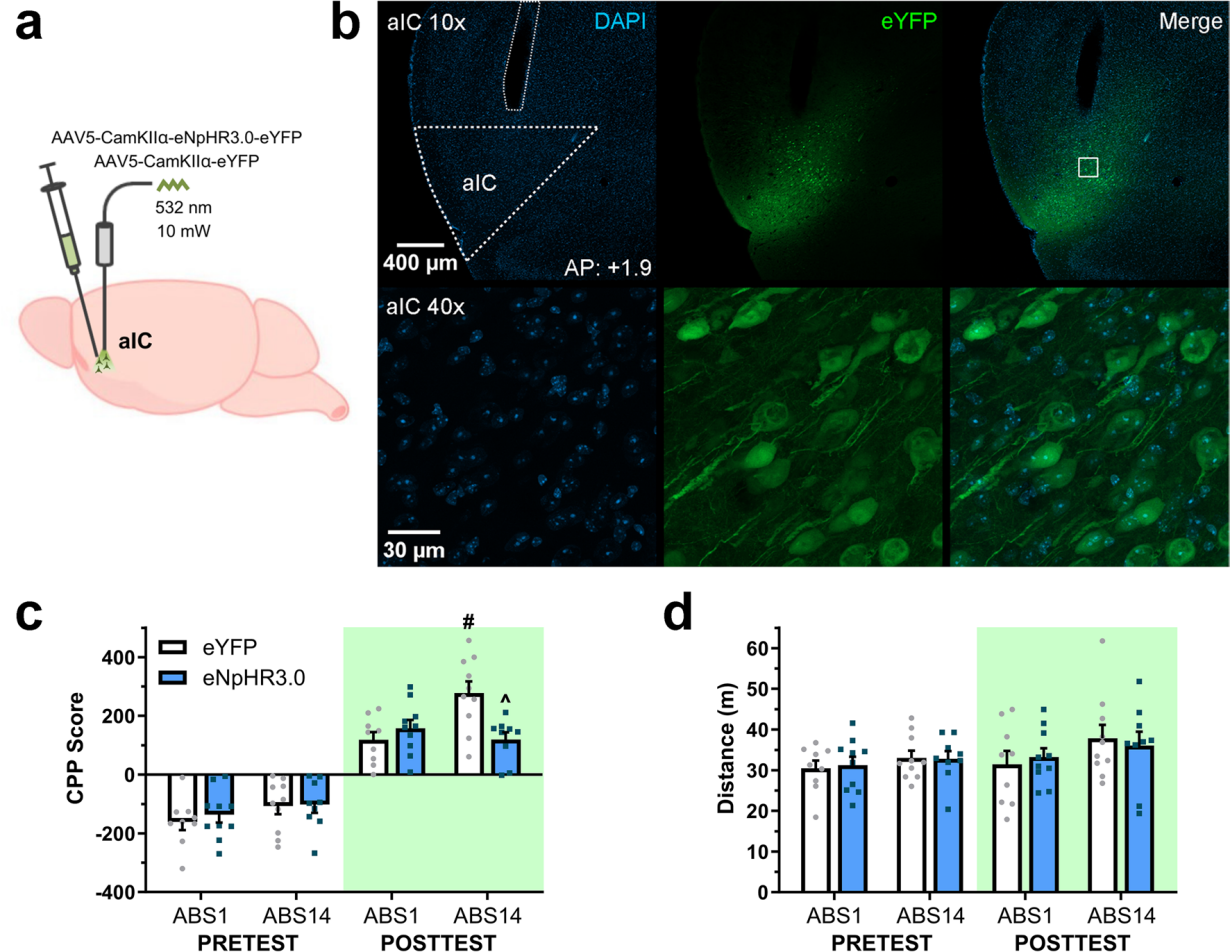
aIC photoinhibition reduces the abstinence-exacerbated CPP expression. **a** Schematic of vector delivery and aIC photoinhibition. **b** Representative images of opsin expression in aIC. **c** Baseline preference and CPP expression following early or late abstinence. #*p* < 0.001 vs. eYFP ABS1, ^*p* < 0.001 vs. eYFP ABS14. **d** Motor activity during place preference tests

To assess whether aIC activity affects CPP expression following different abstinence lengths, subjects were bilaterally infused with a virus encoding eNpHR3.0 or eYFP alone into the aIC. We conducted the previously described amphetamine-induced CPP procedure and delivered 532 nm photoinhibition to aIC during a cue-exposure test 1 or 14 days after the last acquisition session (Fig. 2a, b).

By comparing CPP scores between baseline preference and cue-exposure test (Fig. 2c), three-way mixed ANOVA yielded statistically significant main effects of Conditioning (*F*_(1, 34)_ = 217.80, *p* < 0.001) and Abstinence (*F*_(1, 34)_ = 6.10, *p* < 0.05), but not for Group (*F*_(1, 34)_ = 1.09, *p* = 0.30). Moreover, multiple significant interaction effects were detected between Conditioning by Group by Abstinence (*F*_(1, 34)_ = 4.95, *p* < 0.05) and Group by Abstinence (*F*_(1, 34)_ = 6.56, *p* < 0.05). By comparing posttest CPP scores, two-way factorial ANOVA found no significant main effects for Group (*F*_(1, 34)_ = 3.74, *p* = 0.06) or Abstinence (*F*_(1, 34)_ = 3.92, *p* = 0.05) but revealed a significant interaction between these factors (*F*_(1, 34)_ = 10.43, *p* < 0.01). Bonferroni multiple comparison analysis showed significantly higher CPP scores on ABS14 compared to ABS1 for eYFP control groups (*p* < 0.001). However, no such differences were observed between the eNpHR3.0 groups. Additionally, while both groups exhibited similar CPP scores on ABS1, aIC photoinhibition significantly reduced place preference on ABS14 (*p* < 0.001) compared to eYFP controls.

Regarding motor activity during tests (Fig. 2d), three-way mixed ANOVA found no significant main effects for Conditioning (*F*_(1, 34)_ = 3.07, *p* = 0.08), Group (*F*_(1, 34)_ = 0.00, *p* = 0.94), Abstinence (*F*_(1, 34)_ = 2.79, *p* = 0.10), or any interaction between these factors. Three-way mixed ANOVA on motor activity during acquisition (Fig. S2c) showed a statistically significant main effect of the Pairing session (*F*_(1, 34)_ = 36.72, *p* < 0.001), but no significant main effects of Group (*F*_(1, 34)_ = 0.51, *p* = 0.47), Abstinence (*F*_(1, 34)_ = 0.86, *p* = 0.35), or any interaction between these factors.

These results indicate that all groups displayed higher CPP scores after conditioning than their initial preference. Moreover, while abstinence-exacerbated CPP expression was observed for control conditions, aIC photoinhibition significantly reduced CPP expression after 14 days but did not impair expression after one day of abstinence (Fig. 2c). As previously described, chronic exposure to amphetamine produced similar motor sensitization between groups (Fig. S2c), and no differences in motor activity were observed between tests or groups (Fig. 2d).

### aIC-AMY photoinhibition on abstinence-exacerbated CPP expression

**Fig. 3:**
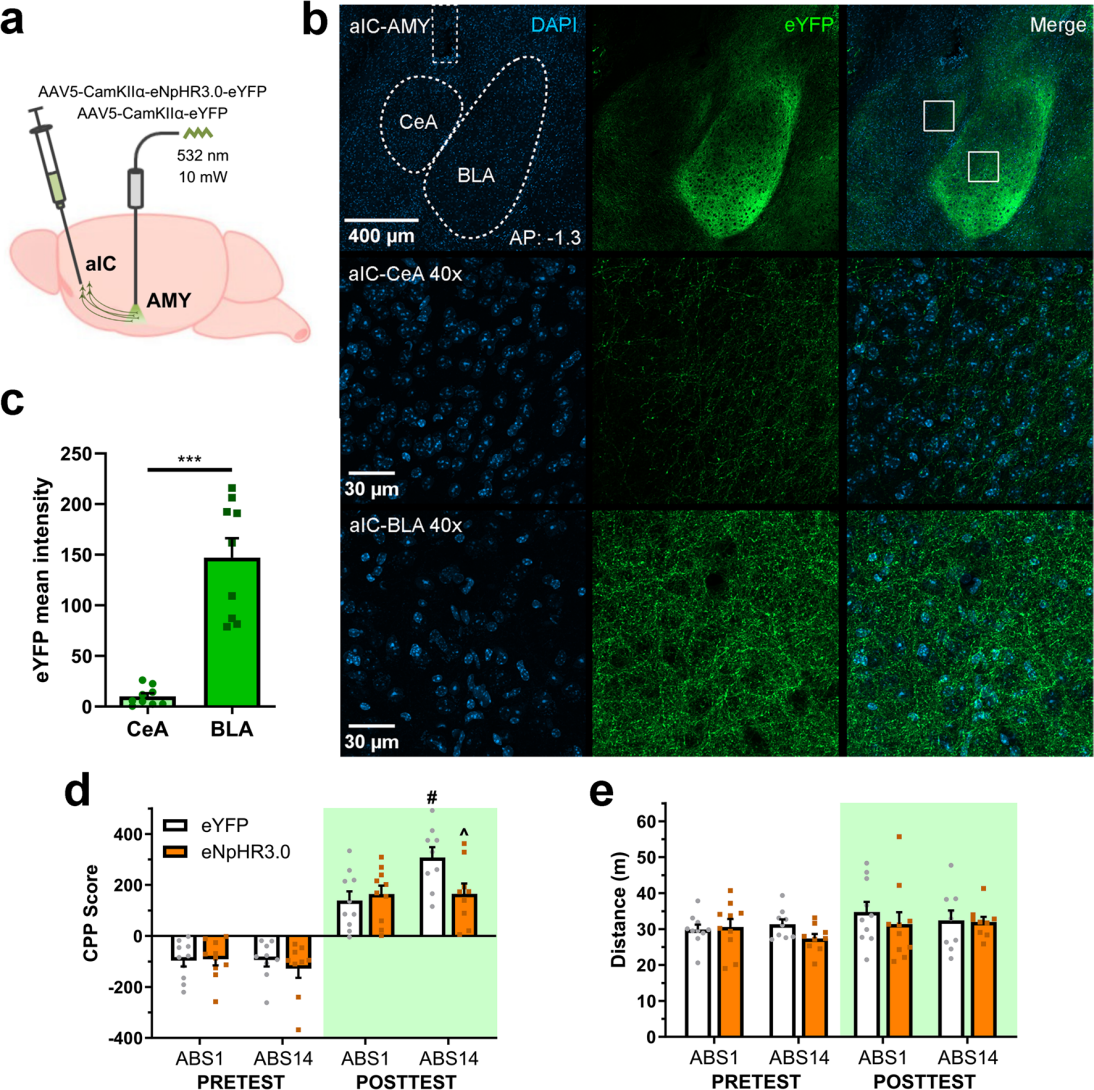
aIC-AMY photoinhibition reduces the abstinence-exacerbated CPP expression. **a** Schematic of vector delivery and aIC-AMY photoinhibition. **b** Representative images of opsin expression in aIC-AMY projection. **c** Mean intensity of aIC projections to CeA and BLA. ***t-test *p* < 0.001 **d** Baseline preference and CPP expression following early or late abstinence. #*p* < 0.01 vs. eYFP ABS1, ^*p* < 0.05 vs. eYFP ABS14. **e** Motor activity during place preference tests

To further assess whether aIC-AMY glutamatergic projection modulates CPP expression following different abstinence lengths, subjects were bilaterally infused with eYFP or eNpHR3.0 vectors into the aIC and followed the amphetamine-induced CPP procedure. Photoinhibition was delivered to AMY during a cue-exposure test held after the same periods (Fig. 3a). A quantitative analysis was performed to measure aIC-AMY projection magnitude between CeA and BLA. Although eYFP positive axons were detected on both nuclei (Fig. 3b, c), the mean intensity of aIC axonic projections was significantly higher on BLA compared to CeA (*t*_(8)_ = 8.04, *p* < 0.001).

By comparing prior and after conditioning CPP scores (Fig. 3d), three-way mixed ANOVA resulted in statistically significant main effects of Conditioning (*F*_(1, 34)_ = 246.67, *p* < 0.001), but not for Group (*F*_(1, 34)_ = 1.87, *p* = 0.18) or Abstinence (*F*_(1, 34)_ = 1.56, *p* = 0.21). Moreover, a significant interaction effect was detected between Conditioning by Abstinence (*F*_(1, 34)_ = 7.30, *p* < 0.05). Two-way factorial ANOVA performed for posttest CPP scores showed a significant main effect of Abstinence (*F*_(1, 34)_ = 5.12, *p* < 0.05) and a significant interaction between Group by Abstinence (*F*_(1, 34)_ = 5.05, *p* < 0.05), while no main effect of Group was detected (*F*_(1, 34)_ = 2.48, *p* = 0.12). Bonferroni multiple comparison analysis showed significantly higher CPP scores on ABS14 (*p* < 0.01) than ABS1 for eYFP control groups. Nevertheless, no such differences were observed between the eNpHR3.0 groups. Additionally, while both groups expressed similar scores on ABS1, aIC-AMY photoinhibition significantly reduced place preference on ABS14 (*p* < 0.05).

When comparing motor activity during tests (Fig. 3e), three-way mixed ANOVA found no significant main effects for Conditioning (*F*_(1, 34)_ = 3.89, *p* = 0.05), Group (*F*_(1, 34)_ = 1.03, *p* = 0.31), Abstinence (*F*_(1, 34)_ = 0.25, *p* = 0.61), or any interaction between these factors. Three-way mixed ANOVA on motor activity during acquisition (Fig. S2d) showed a statistically significant main effect of the Pairing session (*F*_(1, 34)_ = 33.49, *p* < 0.001), but no significant main effects of Group (*F*_(1, 34)_ = 0.04, *p* = 0.83), Abstinence (*F*_(1, 34)_ = 1.35, *p* = 0.25), or any interaction between these factors.

These results indicate that all groups exhibited higher CPP scores after conditioning than their initial preference. Moreover, while abstinence-exacerbated CPP expression was observed in control conditions, aIC-AMY photoinhibition significantly reduced CPP expression after 14 days of abstinence, but not after one day (Fig. 3d). Groups developed similar motor sensitization (Fig. S2d), and no differences in motor activity were observed between tests or groups (Fig. 3e).

### aIC NMDAR antagonism on abstinence-exacerbated CPP expression

**Fig. 4:**
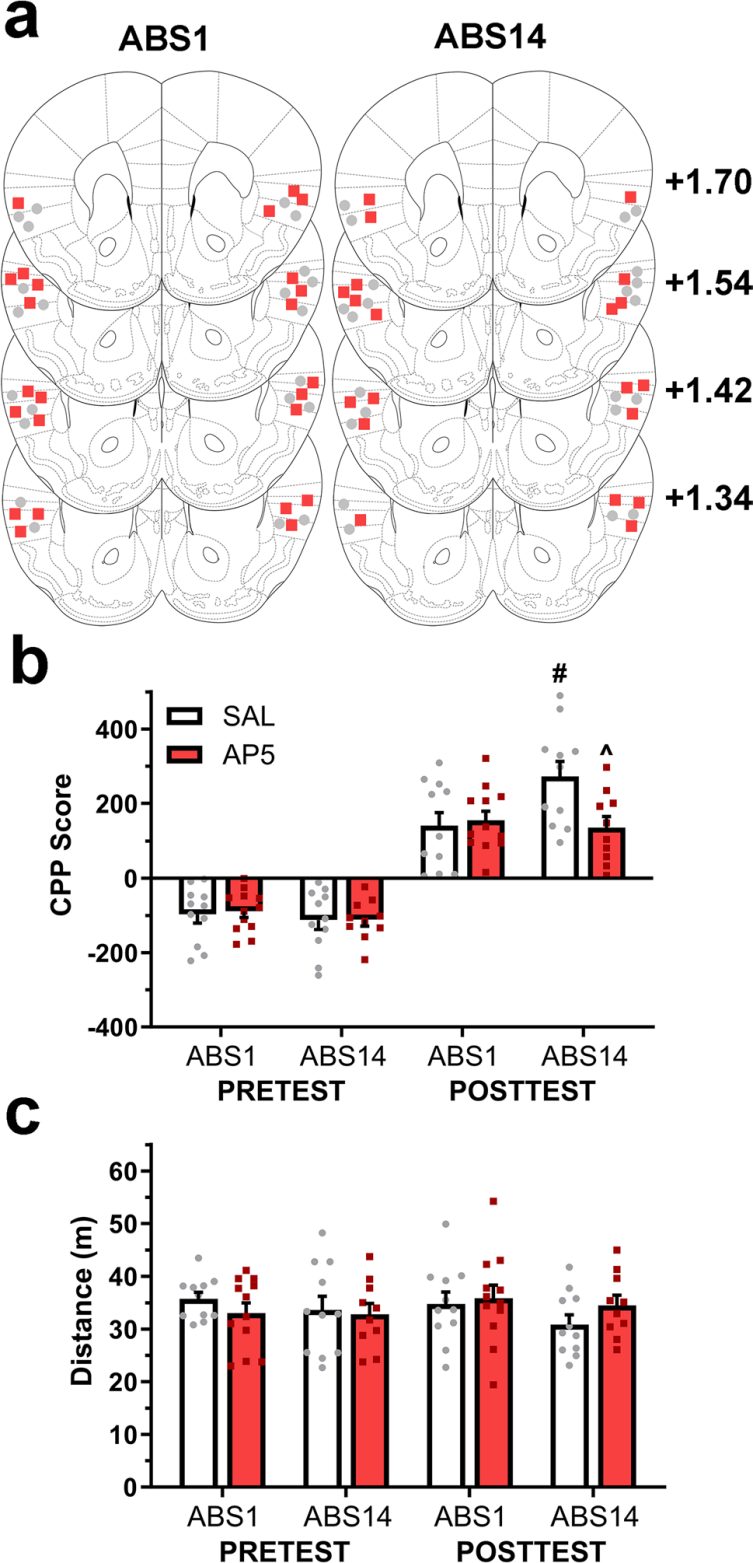
aIC NMDAR antagonism reduces the abstinence-exacerbated CPP expression. **a** Schematic representation of cannulae end traces. Numbers adjoining each coronal section refer to distances from bregma (adapted from Paxinos and Watson, 2008). Circles, SAL; squares, AP5. **b** Baseline preference and CPP expression following early or late abstinence. #*p* < 0.01 vs. SAL ABS1, ^*p* < 0.01 vs. SAL ABS14. **c** Motor activity during place preference tests

To assess whether aIC NMDAR signaling participates in CPP expression following different abstinence periods, subjects were bilaterally implanted with guide cannulae above aIC (Fig. 4a). Then, we conducted the amphetamine-induced CPP procedure and microinjected NMDAR antagonist AP5 or saline into aIC before a cue-exposure test performed at the same abstinence periods.

By comparing pre- and post-conditioning CPP scores (Fig. 4b), three-way mixed ANOVA showed statistically significant main effects of Conditioning (*F*_(1, 40)_ = 200.67, *p* < 0.001), but not for Group (*F*_(1, 40)_ = 2.16, *p* = 0.14), or Abstinence (*F*_(1, 40)_ = 0.93, *p* = 0.33). Moreover, a significant interaction effect was detected between Group by Abstinence (*F*_(1, 40)_ = 4.23, *p* < 0.05). Two-way factorial ANOVA performed for posttest CPP scores showed no significant main effects for Group (*F*_(1, 40)_ = 3.45, *p* = 0.07), or Abstinence (*F*_(1, 40)_ = 2.91, *p* = 0.09) but revealed a significant interaction between these factors (*F*_(1, 40)_ = 5.29, *p* < 0.05). Bonferroni multiple comparison analysis showed significantly higher CPP scores on ABS14 (*p* < 0.01) compared to ABS1 for saline groups. However, no such differences were observed between AP5 groups. Additionally, while both groups expressed similar scores on ABS1, NMDAR antagonism significantly reduced place preference for ABS14 (*p* < 0.01).

Three-way mixed ANOVA on motor activity during preference tests (Fig. 4c) found no significant main effects for Conditioning (*F*_(1, 40)_ = 0.02, *p* = 0.86), Group (*F*_(1, 40)_ = 0.02, *p* = 0.86), Abstinence (*F*_(1, 40)_ = 1.16, *p* = 0.28), or any interaction between these factors. Regarding motor activity during acquisition (Fig. S2e), three-way mixed ANOVA resulted in a statistically significant main effect of the Pairing session (*F*_(1, 40)_ = 73.08, *p* < 0.001), but no significant main effects of Group (*F*_(1, 40)_ = 0.00, *p* = 0.95), Abstinence (*F*_(1, 40)_ = 0.12, *p* = 0.72), or any interaction between these factors.

These results indicate that all groups displayed higher CPP scores after conditioning compared to their baseline preference. Moreover, while abstinence-exacerbated CPP expression was observed in control groups, aIC NMDAR antagonism significantly reduced CPP expression after 14 days but did not affect the expression after a single day of abstinence (Fig. 4b). During acquisition, motor sensitization developed due to chronic amphetamine treatment, with no differences between groups or abstinence periods (Fig. S2e), and no differences in motor activity were observed between tests or groups (Fig. 4c).

## Discussion

In the present study, we described an abstinence-exacerbated pattern of CPP expression between 1 and 14 days of amphetamine withdrawal. Our results provide behavioral evidence of the incubation of amphetamine craving on a Pavlovian-based CPP procedure. Moreover, optogenetic photoinhibition of either aIC or aIC-AMY projection significantly reduced the abstinence-exacerbated CPP expression after 14 days of withdrawal. Meanwhile, these treatments did not produce any effect on the day following the last amphetamine exposure. Similarly, pharmacological blockade of aIC NMDAR also attenuated CPP expression following 14 days of abstinence, while it had no effect in the 1-day abstinence group.

CPP expression constitutes a memory-evoked set of approach behaviors that reflect the secondary motivational properties of contextual cues, which are consequently acquired through contiguous pairing with the primary rewarding properties of unconditioned stimuli (Huston et al. 2013; McKendrick and Graziane 2020; Tzschentke 2007). With psychoactive substances, the magnitude of CPP expression depends upon the drug and dose used, apparatus and design bias, the number and length of conditioning sessions, and the activity of brain circuits that control memory and motivation processing (Bardo and Bevins 2000; McKendrick and Graziane 2020; Tzschentke 2007). To our knowledge, only a couple of studies have previously described the emergence of an abstinence-exacerbated CPP expression induced by morphine (Li et al. 2008), cocaine (Calcagnetti and Schechter 1993), and MDMA (Daza-Losada et al. 2007). This pattern of anticipatory approach responses reflects an enhancement of the motivational value of drug-related contextual cues following withdrawal, as consistently observed in self-administration models (Altshuler et al. 2020; Pickens et al. 2011; Wolf 2016). Although we attempted to counter the disproportionate CPP expression through a non-contingent amphetamine dose administered 24 h before the late abstinence test, no significant attenuation was observed. Such an outcome suggests that some of the underlying alterations that occur during the first weeks of abstinence and consequently heighten the motivational value of drug-related stimuli may not be reversed by recent drug exposure, indicating an enduring relapse vulnerability that relies on associative learning and a time-dependent maladaptive enhancement of motivated behavior.

### Insular cortex role on drug-related contextual memory

Behavioral pharmacology studies indicate that insular cortex activity regulates CPP expression. After 10 days of forced abstinence, the inactivation of voltage-gated Na^+^ channels within the posterior insular cortex (pIC) abolishes amphetamine-induced CPP expression (Contreras et al. 2007), suggesting an impairment of contextual memory retrieval. A separate study determined that pIC cholinergic M_1_/M_4_ signaling regulates the magnitude of morphine-induced CPP expression during early abstinence (Wu et al. 2014). Collectively, these studies indicate that insula function is not only involved in the retrieval of drug-related contextual memory but also suggests a role in disproportionate drug seeking.

It has been noted that CPP studies on aIC have mainly focused on the acquisition (Li et al. 2013; Loney et al. 2021; Scott and Hiroi 2011) and memory extinction (Contreras et al. 2012; Gil-Lievana et al. 2020), but its specific involvement in CPP expression has yet to be addressed. Our study found that blocking aIC NMDAR or photoinhibition of aIC pyramidal neurons significantly reduced the expression of CPP during late but not early amphetamine withdrawal. These results suggest that aIC mediates the enhancement of context-elicited drug seeking following abstinence, rather than contextual memory retrieval. The disparity observed between our findings and previous studies may be attributable to technical variations, the most relevant being the withdrawal length before evaluating place preference. However, it could also point to a distinct contribution from insular cortex regions to contextual memory recall. Evidence supporting this hypothesis is provided by the observation that inhibition of an intermediate region of the insula reduces, but does not abolish, the expression of morphine-induced CPP during early abstinence (Zhang et al. 2019). Moreover, our group has previously reported that NMDAR signaling within the insula does not mediate contextual memory retrieval on a Morris water maze (Gutiérrez et al. 1999) and that BLA glutamatergic input to aIC is not necessary for contextual memory retrieval, although accelerated the extinction of CPP induced by photostimulation of VTA tyrosine hydroxylase-expressing neurons (Gil-Lievana et al. 2020).

Our findings generally align with those from self-administration studies that examined aIC involvement in contextual and discrete cue-induced reinstatement of drug seeking. The inactivation of aIC (Arguello et al. 2017; Campbell et al. 2019; Cosme et al. 2015; Di Pietro et al. 2006; Ghareh et al. 2022; Pushparaj et al. 2015) attenuates reinstatement following the extinction of operant responding during a period of drug unavailability or punishment. In this manner, aIC activity may mediate the motivational reactivity to drug-related stimuli following abstinence and ultimately influence the magnitude of drug seeking upon re-exposure.

### Insula and incubation of drug craving

It has been proposed that the incubation of drug craving stems from structural modifications that progressively accumulate within the brain reward circuit (Altshuler et al. 2020; Pickens et al. 2011; Wolf 2016). These may constitute compensatory adaptations in response to the sudden discontinuation of chronic drug exposure, functionally leading to enhanced synaptic transmission and neuronal reactivity of structures that receive afferents from the mesocorticolimbic dopamine system. Some examples that develop during abstinence from cocaine or methamphetamine self-administration are the progressive upregulation of brain-derived neurotrophic factor in VTA, NAc, and amygdala (Grimm et al. 2003), the internalization of membrane metabotropic glutamate type 1 receptors followed by the synaptic insertion of Ca^2+^ permeable AMPA receptors in medium spiny neurons of NAc core (Conrad et al. 2008; Loweth et al. 2014; Scheyer et al. 2016), and the functional recruitment of target structures, such as CeA (Li et al. 2015b; Lu et al. 2005) and ventral medial prefrontal cortex (Koya et al. 2009). The involvement of the insular cortex in the incubation of drug craving was initially proposed due to an abstinence-dependent correlation between the magnitude of cue-induced nicotine seeking and evidence of increased activity of protein kinase A within aIC (Abdolahi et al. 2010). Further research showed that pharmacological inactivation of aIC reduced cue-induced methamphetamine or fentanyl seeking following voluntary abstinence achieved through a simple-choice procedure that relied on simultaneous food availability (Reiner et al. 2020; Venniro et al. 2017). Moreover, CeA glutamatergic afferents from aIC were found to be partially responsible for the magnitude of cue-induced methamphetamine seeking following voluntary abstinence (Venniro et al. 2017). However, assessment during early abstinence was not performed on these conditions. Our findings extend those reported in the literature during late abstinence and further indicate that CPP expression during early amphetamine abstinence is not mediated by the activity of aIC and its transmission to the amygdala.

### aIC-AMY neuronal circuit on cue-induced drug seeking

The insular cortex has been proposed to integrate positive and negative emotional valence signals, on a topological distribution along its anterior-posterior axis (Gehrlach et al. 2019; Peng et al. 2015). Accordingly, aIC and pIC display a gradient of anatomical projections to amygdala nuclei, corresponding to BLA and CeA (Nicolas et al. 2023; Wang et al. 2018). In agreement with these studies, our results show that aIC axonic projections were found in both nuclei but innervate BLA more extensively than CeA. Extensive research has established a critical role for BLA in mediating associative relationships between drug-related stimuli and seeking behaviors. Although BLA inactivation has been shown to reduce drug-induced CPP expression (Hsu et al. 2002), this effect has not been consistently observed (Fuchs et al. 2002; Zarrindast et al. 2004), perhaps due to functional differences between BLA subregions (Kantak et al. 2002). Our results indicate that aIC-AMY projection activity does not mediate CPP expression on early abstinence but becomes recruited during late abstinence. Consequently, the activity of BLA neurons innervated by aIC may modulate the magnitude of conditioned drug seeking following withdrawal. This interpretation concurs with studies examining the involvement of BLA in contextual and discrete cue-induced reinstatement of cocaine seeking, wherein BLA inactivation has been described to reduce reinstatement following extinction (Fuchs et al. 2005; Kantak et al. 2002; McLaughlin and See 2003; Pelloux et al. 2018). Moreover, although BLA is generally not considered to participate in the incubation of drug craving (Li et al. 2015b; Li et al. 2008; Lu et al. 2005), evidence indicates that incubation of cocaine craving is partially mediated by time-dependent adaptations at NAc synapses that receive glutamatergic inputs from BLA (Lee et al. 2013).

Alternately, during forced abstinence critical adaptations develop within CeA neurons and ultimately mediate the incubation of drug craving. Cue exposure following prolonged abstinence leads to disproportioned cocaine seeking and morphine-induced CPP expression, mediated by NMDAR signaling and extracellular signal-regulated kinase (ERK) phosphorylation within CeA (Li et al. 2008; Lu et al. 2005). Conversely, CeA NMDAR agonism during early abstinence promotes an ERK-dependent enhancement of cue-induced cocaine seeking (Lu et al. 2005) and a heightened expression of morphine-induced CPP (Li et al. 2008; Rezayof et al. 2007). Moreover, CeA inactivation reduces cue-induced methamphetamine seeking after prolonged, but not early withdrawal (Li et al. 2015b). This evidence indicates a modulatory influence of CeA activity in motivated behavior, possibly due to its involvement in the allocation and enhancement of incentive salience to reward-related cues (Warlow and Berridge 2021). Overall, our results suggest a role for aIC in the attribution of disproportionate motivational value to amphetamine-related contextual cues following late abstinence, partially through BLA and CeA projections. Although the activity of somatostatin-expressing neurons within CeA lateral nucleus has already been related to enhanced cue-induced drug seeking following prolonged methamphetamine withdrawal (Venniro et al. 2018, 2020), a detailed functional assessment between specific BLA and CeA neuronal subpopulations that receive input from aIC remains to be explored.

### Limitations

It is known that behavioral assessment through halorhodopsin-based optogenetic photoinhibition requires continuous light delivery during the evaluation length, which typically extends for several minutes. This period may hold some technical constraints, including the gradual hampering of inhibition efficiency, and unspecific effects due to thermal increase (Wiegert et al. 2017). To this end, the illumination parameters used in the current study have been previously shown to reduce neurotransmitter release and modulate the electrophysiological activity in opsin-expressing target structures (Gálvez-Márquez et al. 2022; Gil-Lievana et al. 2020). However, control subjects exposed to the same conditions do not exhibit changes in these measures. Similarly, in this study, we found a specific CPP reduction at 14 days, but not after a single day, in both aIC and aIC-AMY experiments, without any observable modification in motor activity measured by the traveled distance during the light exposure, suggesting no evidence of unspecific effects by photoinhibition.

Moreover, our findings suggest that aIC plays a functional role in enhancing the motivational value of drug-related contextual cues after prolonged abstinence. However, we cannot rule out the possibility that progressive adaptations within this region may result in a potential involvement in retrieving contextual memories, as an alternative explanation. Nonetheless, we did not observe that CPP expression was impaired during early abstinence, suggesting that this brain circuit does not mediate contextual memory recall. In addition, it is important to note that the extent to which these insights apply to the female population remains unexplored. In this regard, existing evidence indicates that the incubation effect occurs in both sexes. Even though the incubation of cocaine craving may be more pronounced in females than males (Nicolas et al. 2022), specific experimental conditions appear pertinent.

### Translational relevancy to SUDs treatment

The selective targeting of insula activity holds significant implications for the clinical treatment of SUDs. Functional imaging studies performed on experienced people who use drugs have shown that exposure to drug-associated cues activates the insular cortex (Kühn and Gallinat 2011). Notably, a positive correlation has been described between insula reactivity and drug craving during cue exposure (Bonson et al. 2002; Kühn and Gallinat 2011; Luijten et al. 2011; Wang et al. 2007). Furthermore, individuals with robust insula reactivity to drug-associated cues are more vulnerable to relapse (Janes et al. 2010). These observations suggest that the insula processes drug-related stimuli and may influence relapse by integrating subjective drug craving. Clinical studies employing deep repetitive transcranial magnetic stimulation to modulate insula activity have yielded promising results for tobacco use disorder treatment (Dinur-Klein et al. 2014; Ibrahim et al. 2019; Zangen et al. 2021).

## Conclusion

The present study described an abstinence-exacerbated pattern of CPP expression following the first weeks of amphetamine withdrawal, providing behavioral evidence of the incubation of amphetamine craving through a Pavlovian-based associative paradigm. Photoinhibition performed during late abstinence contextual re-exposure indicates that the abstinence-exacerbated CPP expression depends on the intact activity of aIC glutamatergic pyramidal neurons and their projection to amygdala nuclei. Moreover, along with amphetamine withdrawal, enhanced CPP expression becomes gradually dependent upon NMDAR-mediated glutamatergic signaling within aIC. Conversely, the previous manipulations during early abstinence did not affect CPP expression, suggesting that activation of aIC and its output to the amygdala does not mediate contextual memory retrieval but modulates the motivational value of amphetamine-related contextual cues. This evidence indicates that aIC and its glutamatergic projection to the amygdala constitute critical neurobiological underpins for the incubation of amphetamine craving. Furthermore, aIC may be a suitable therapeutic target to treat drug craving and relapse precipitated by learned associations during a specific time window where abstinence length should be thoroughly considered.

### Supplementary information

ESM 1

## Data Availability

Analyzed data will be made available upon reasonable request to the corresponding author.

## Abbreviations

aIC: Anterior insular cortex
aIC-AMY: Anterior insular cortex projection to the amygdala
BLA: Basolateral nucleus of the amygdala
CeA: Central nucleus of the amygdala
CPP: Conditioned place preference
eNpHR3.0: Halorhodopsin
eYFP: Enhanced yellow fluorescent protein
NAc: Nucleus accumbens
SUDs: Substance use disorders
VTA: Ventral tegmental area
