# Supplementary material for: The anterior insula and its projection to amygdala nuclei modulate the abstinence-exacerbated expression of conditioned place preference
Source: Psychopharmacology (Berl). 2023 Nov 27;241(3):445–59. doi: 10.1007/s00213-023-06499-0 (PMC10884150; doi:10.1007/s00213-023-06499-0)
Supplement: Supplementary file 1 — ESM 1 [file 213_2023_6499_MOESM1_ESM.pdf]

The anterior insula and its projection to amygdala nuclei modulate the abstinence-exacerbated expression of conditioned place preference. *Psychopharmacology*

Agoitia A., Cruz-Sanchez A., Balderas I., & Bermúdez-Rattoni F.

Supplementary Information

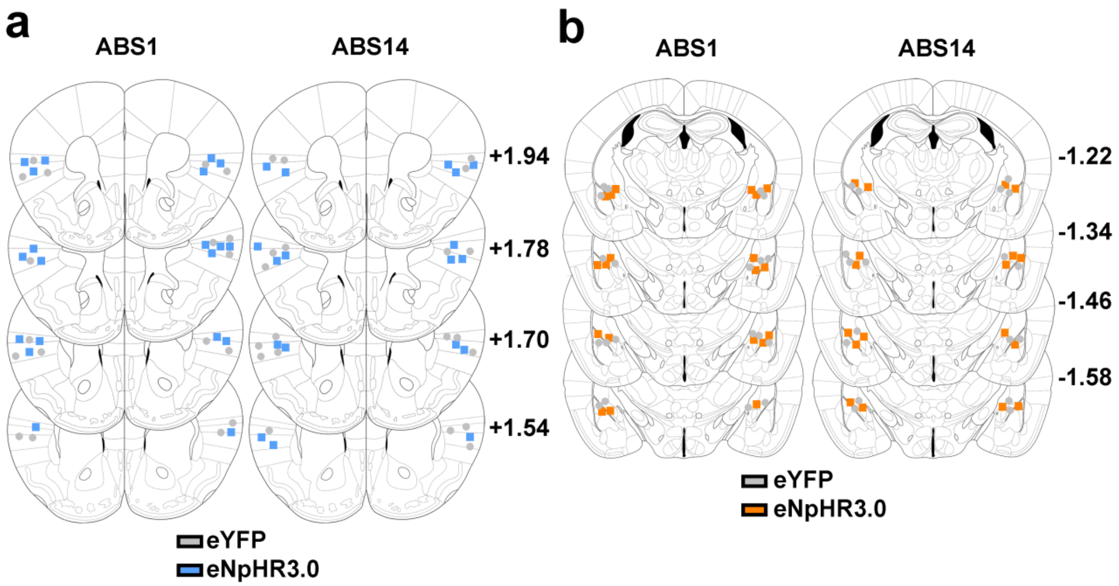

Fig. S1 Schematic representation of the optical fiber end traces of: **a** aIC photoinhibition, **b** aIC-AMY photoinhibition. Numbers adjoining each coronal section refer to distances from bregma (adapted from Paxinos and Watson, 2008). Circles; eYFP, squares; eNpHR3.0

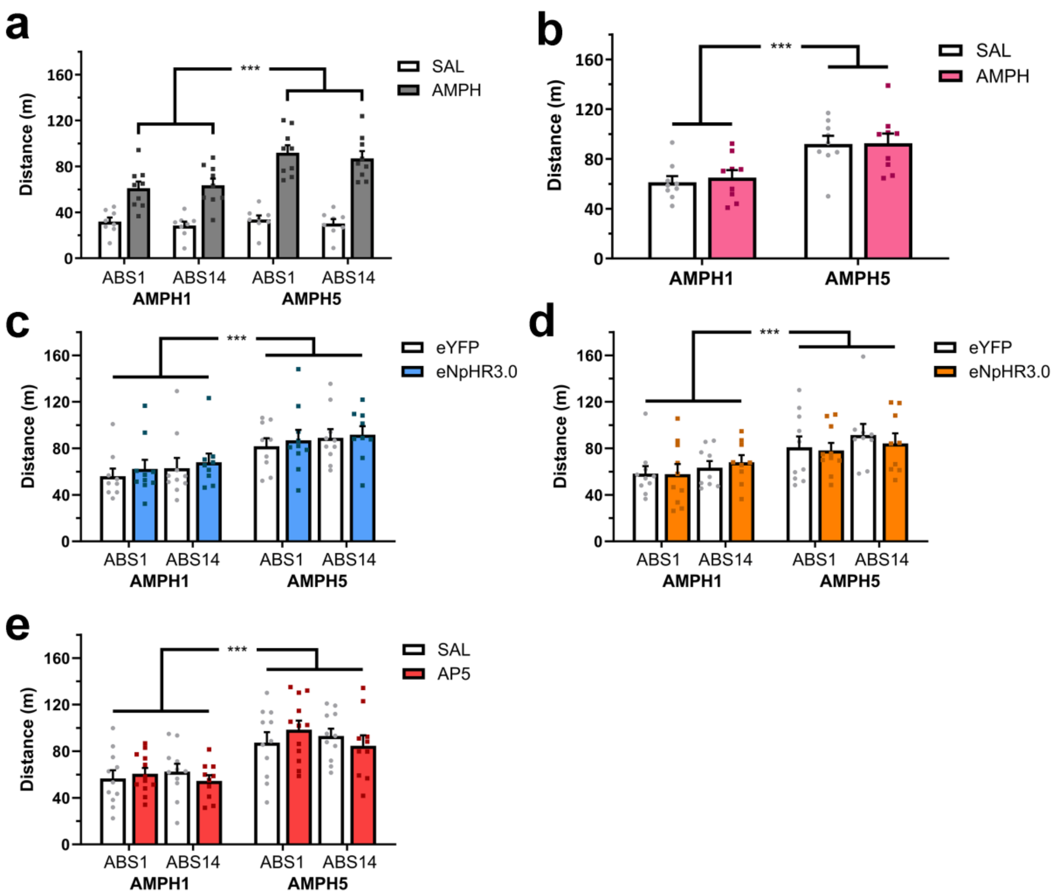

Fig. S2 Motor sensitization following chronic amphetamine treatment. Motor activity during first (AMPH1) and last (AMPH5) amphetamine exposure during CPP acquisition of: **a** saline or amphetamine-treated subjects, **b** saline or amphetamine exposure 24h before late abstinence CPP expression, **c** aIC photoinhibition, **d** aIC-AMY photoinhibition, **e** aIC NMDAR antagonism. ***; 2- or 3-way mixed ANOVA main effect p<0.001

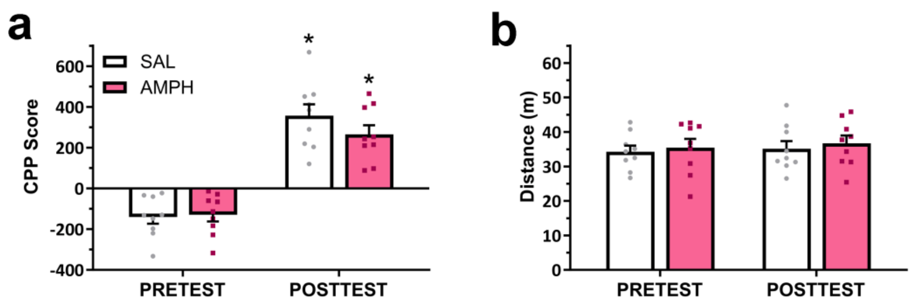

Fig. S3 Recent amphetamine exposure does not counter abstinence-exacerbated CPP expression. **a** Baseline preference and CPP expression following late abstinence. ***; 2-way mixed ANOVA main effect p<0.001 **b** Motor activity during place preference tests.
